# A178 GASTROINTESTINAL STRICTURES IN A PEDIATRIC PATIENT WITH SATOYOSHI SYNDROME

**DOI:** 10.1093/jcag/gwad061.178

**Published:** 2024-02-14

**Authors:** K Pohoreski, K Pajunen, M Brundler, I Wrobel

**Affiliations:** University of Calgary, Calgary, AB, Canada; University of Calgary, Calgary, AB, Canada; University of Calgary, Calgary, AB, Canada; University of Calgary, Calgary, AB, Canada

## Abstract

**Background:**

Satoyoshi syndrome (SS) is a rare, progressive, multisystem disease including muscle spasms, alopecia, skeletal deformities, and diarrhea, with suspected autoimmune etiology. Treatment includes corticosteroids, immunoglobulin therapy, or immunosuppression. Reported endoscopic findings include loss of intestinal folds and nodularity in the stomach and the intestine, with histology showing gastritis cystica polyposa and lymphoplasmacytic infiltrate, as well as fibrosis noted on autopsy reports. To date, no cases of intestinal stricture have been described in SS.

**Aims:**

To describe an original case of intestinal strictures in SS.

**Methods:**

Case report and literature review.

**Results:**

We present a case of a 10-year-old girl with chronic diarrhea and intermittent hematochezia, postprandial abdominal pain, intermittent non-bilious emesis, stunted growth, and weight loss, with acquired alopecia and severe muscle cramps. Examination showed a non-dysmorphic, small child with alopecia totalis and a distended, tender abdomen. Investigations demonstrated an unremarkable endocrine work-up, mild hypoalbuminemia, microcytic anemia, and anti-transglutaminase serology at 3-4x ULN with no improvement on the gluten-free diet. Endoscopy identified a duodenal stricture with a very distended stomach and duodenal bulb, mucosal nodularity and bridging creating a web-like appearance, and edema and friability of the left-sided colon. MR enterography confirmed an isolated duodenal stricture. Pathology displayed patchy fibrosis with crypt/glandular dilatation in the antrum, duodenum, and rectum. Skeletal imaging detected physeal widening and sclerosis with mild slipping of the epiphyses. Auto-immune markers included positive ENA profile for anti-Sm, anti-Sm/RNP, and anti-RNP-A. The patient was treated with corticosteroids in addition to a series of endoscopic dilatations for duodenal stricture with symptomatic benefit, with addition of upadacitinib for alopecia management. Follow-up revealed intermittent worsening of symptoms with steroid taper, necessitating further duodenal dilatations, and evidence of a new anal stricture. The patient remains on corticosteroid therapy with ongoing surveillance.

**Conclusions:**

This case identifies the first report of intestinal strictures in SS, which may suggest that in addition to suspected autoimmune pathogenesis, disorders of fibrogenesis may need to be considered.

**Funding Agencies:**

None

